# Application of metabolomics in prediction of lymph node metastasis in papillary thyroid carcinoma

**DOI:** 10.1371/journal.pone.0193883

**Published:** 2018-03-06

**Authors:** Ji Won Seo, Kyunghwa Han, Jandee Lee, Eun-Kyung Kim, Hee Jung Moon, Jung Hyun Yoon, Vivian Youngjean Park, Hyeon-Man Baek, Jin Young Kwak

**Affiliations:** 1 Department of Radiology, Severance Hospital, Research Institute of Radiological Science Yonsei University College of Medicine, Seoul, Republic of Korea; 2 Department of Radiology, Severance Hospital, Research Institute of Radiological Science and Center for Clinical Imaging Data Science, Yonsei University College of Medicine, Seoul, Republic of Korea; 3 Department of Surgery, Yonsei University College of Medicine, Seoul, Republic of Korea; 4 Gachon University, Department of Biomedical Engineering, Incheon, Republic of Korea; University of South Alabama Mitchell Cancer Institute, UNITED STATES

## Abstract

**Purpose:**

The aim of this study was to find useful metabolites to predict lymph node (LN) metastasis in patients with papillary thyroid cancer (PTC) through a metabolomics approach and investigate the potential role of metabolites as a novel prognostic marker.

**Materials and methods:**

Fifty-two consecutive patients (median age: 41.5 years, range 15–74 years) were enrolled who underwent total thyroidectomy and central LN dissection with or without lateral LN dissection in Severance Hospital between October 2013 and July 2015. The study specimens were provided by the Severance Hospital Gene Bank, and consisted of PTC from each patient. The specimens were prepared for proton nuclear magnetic resonance (^1^H-NMR) spectroscopy. Spectral data by ^1^H-NMR spectroscopy were acquired, processed, and analyzed. Patients were grouped in three ways, according to the presence of LN metastasis, central LN metastasis and lateral LN metastasis. Chi-square test and the student t-test were used to analyze categorical variables and continuous variables, respectively. The Mann-Whitney U test was used for univariate analysis of metabolites. Orthogonal projections to latent structure discriminant analysis (OPLS-DA) was used for multivariate analysis to discriminate metabolic differences between the two groups.

**Results:**

Among 52 patients, 32 had central LN metastasis and 19 had lateral LN metastasis. No clinical or histopathological characteristic was significantly different for all comparisons. On univariate analysis, no metabolite showed significant difference for all comparisons. On multivariate analysis, OPLS-DA did not discriminate the presence and absence of LN metastasis. Lactate was found to be the most promising metabolite.

**Conclusions:**

No metabolite could discriminate the presence of LN metastasis. However, lactate was found to be the most promising metabolite for discrimination. Further studies with larger sample sizes are needed to elucidate significant metabolites which can indicate the presence of LN metastasis in patients with PTC.

## Introduction

The incidence of thyroid cancer has increased worldwide during the last few decades and it is now the most common endocrine malignancy [1,2]. Papillary thyroid cancer (PTC) is the most common histologic type, accounting for 85% to 90% of thyroid malignancies [3]. Most patients with PTC have excellent prognosis, with the 10-year survival rate being about 90% [3,4]. However, some patient subsets suffer from more aggressive PTC characterized by recurrent disease, lymph node (LN) or distant metastasis [5]. These patients may need more extensive surgery including total thyroidectomy with therapeutic or prophylactic LN dissection and postoperative radioactive iodine (RAI) ablation [6]. Therefore, being able to predict risk is important and would help stratify patients for proper treatment [7].

Several prognostic factors have been discovered including patient age at diagnosis, size and extent of the primary tumor, cervical LN metastasis, and occurrence of distant metastasis [8,9]. Of these prognostic factors, it is LN metastasis that is associated with an increased incidence of recurrence [10]. The incidence of central and lateral LN metastasis has been reported about 50–60% and 4.1–42.6%, respectively depending on the study [11–13]. Ultrasound plays main role in detection and characterization of cervical LN and ultrasound-guided fine-needle aspiration biopsy (FNAB) is the main diagnostic tool for the diagnosis of metastatic cervical LN in patient with PTC [14].However even under ultrasound guidance, approximately 5–10% of the FNAB results of cervical LN might be nondiagnostic and 6–8% might be false negative [15].

Advances in genetic research and molecular biology have discovered several genetic changes behind thyroid cancer [16]. The RAS mutation, RET/PTC rearrangement, and PAX8-peroxiome proliferator-activated receptor γ1 fusion are important oncogenic genetic alterations in thyroid cancer [17–19]. Also, the BRAF^V600E^ mutation results from a single thymine-to-adenosine transversion which is a high specific marker for PTC [7,20]. The BRAF^V600E^ mutation is useful when diagnosing PTC, especially in cases in which the cytologic results only provide suspicious results for PTC [21]. However, the association between the BRAF^V600E^ mutation and LN metastasis remains under question [22–24].

Metabolomics is a new field in biological science, which uses analytic tools in conjunction with pattern recognition approaches and bioinformatics [25]. The metabolome is the final downstream product of gene expression; thus, it reflects changes in the transcriptome and the proteome [26]. Alterations in metabolic processes occur during carcinoma development and progression, along with histologic and cytologic changes [27]. Understanding the biochemistry of cancer may enable the development of powerful diagnostic tools and the identification of new biomarkers [28]. Several studies have proven that the metabolomics approach allows the characterization of different types of malignancies in other organs [29–31]. For example, the presence of 2-hydroxyglutarate which is a metabolite detected by magnetic resonance spectroscopy (MRS) correlated with mutations in isocitrate dehydrogenase 1 or 2 (*IDH1*, *2*) in patients with gliomas of the brain [32]. *IDH1* or *IDH*2 mutation is an significant marker of positive prognosis and chemosensitivity [33]. In patients with breast cancer, the combined magnetic resonance (MR) protocol of dynamic contrast-enhanced MR imaging and proton nuclear magnetic resonance (^1^H-NMR) spectroscopy improved sensitivity and specificity in the diagnosis of breast cancer [30].

Several studies have applied metabolomics to PTC [34,35]. To our knowledge, little is known about the association between metabolomics and the presence of LN metastasis in PTC. The aim of our study was to investigate metabolic differences according to the presence or absence of LN metastasis in patients with PTC in the search for a potential novel prognostic marker.

## Materials and methods

This study was approved by the Institutional Review Board of Severance Hospital, Yonsei University College of Medicine in Seoul, Korea.

### Patients and sample collection

Patients who underwent total thyroidectomy and central LN dissection with or without lateral LN dissection in Severance Hospital between October 2013 and July 2015 were enrolled in this study. The specimens for this study were provided by the Severance Hospital Gene Bank, and consisted of conventional PTC from each patient. All samples were obtained with informed consent under institutional review board-approved protocols. Samples were snap-frozen in liquid nitrogen immediately after surgery and then stored at −70°C. All of the data were securely protected while being made available only to investigators and analyzed anonymously.

### Preparation of tissue extracts

Frozen thyroid samples were finely ground in a mortar under liquid nitrogen. Percholoric acid (4%; 1:4, w/v) was added to each sample, followed by centrifugation at 20000 ɡ for 15 min. The supernatant was transferred to a new tube where chloroform/tri-n-octylamine (78%/22%; v/v) was added in a 1:2 volumetric ratio to increase the pH to ~6. The samples were centrifuged at 20000 ɡ for 15 min. The aqueous phase was removed and transferred to a microfuge tube, and then lyophilized. 200L of deuterium oxide (99.96%; Cambridge Isotope Laboratories) was added to each sample and the pH was adjusted to 7.0 with 0.2-1L of 1M sodium deuteroxide (99.5%; Cambridge Isotope Laboratories). The pH-neutral samples were then centrifuged at 15000 ɡ for 1 min., and the supernatant was then removed and placed in a 3-mm NMR tube for subsequent NMR analysis.

### Proton NMR spectroscopy

^1^H-NMR spectroscopy was performed on a Bruker Advanc spectrometer (Bruker Instruments, Billerica, MA) operating at a proton NMR frequency of 700.40 MHz (16.45 Tesla). A one-dimensional CPMG (Carr-Purcell-Meiboom-Gill) pulse sequence was used to obtain thyroid metabolite profiles with a 90 degree pulse length of about 7 μs. The water signal was suppressed using a selective excitation pulse followed by a pulsed field gradient in the z-axis. The spectral acquisition parameters were as follows: 16K complex data points, 8417 Hz sweep width, 2.0 s acquisition time, 2.0 s relaxation delay, 1.5 s presaturation time (5.5 s total time of repetition (TR)), 1.0 ms interpulse delay (2 ms time of echo (TE)), 32 number of transients, 20.2 receiver gain and total acquisition time of 5 min. An experimental line broadening function of 0.2 Hz and automatic zero-filing of a factor of 2 was applied to each FID prior to Fourier transformation. 1H-NMR spectra were manually corrected for phase and baseline distortion using TOPSPIN 3.5 (Bruker Instruments, Billerica, MA) and referenced to the trimethylsilyl propionic acid (TSP) signal (0.0 ppm).

### Data and statistical analysis

All ^1^H-NMR spectra were processed and analyzed using Chenomx NMR Suite 7.7 software (Chenomx, Edomonton, Canada). Post-processing consisted of Fourier transformation, phasing and baseline correction. Chemical shifts were referenced to TSP at 0.0 ppm. Spectral regions from 0.5 to 9.0 ppm [Isoleucine (Iso), Leucine (Leu), Valine (Val), Threonine (Thr), Lactate (Lac), Alanine (Ala), Uracil (Ura), Lysine (Lys), Glutamate (Glu), Methionine (Met), Aspartate (Asp), Free choline (Cho), Phosphocholine (PC), Glycerophosphocholine (GPC), Taurine (Tau), Myo-inositol (m-Ins), Glycine (Gly), Phosphoethanolamine (PE), Inosine (Ino), Tyrosine (Tyr), Hypoxanthine (Hyp), Formate (For), Succinate (Suc), and Uridine (Uri)] were selected for quantification. The peak amplitudes of the metabolites were measured by fitting a Voigt (e.g., Gauss+Lorentz) line-shape function. Metabolites [mM] were quantified by comparing the integrated TSP signal to the metabolite signal.

Patients were grouped into two groups in three different ways, according to the following factors: the presence or absence of LN metastasis, central LN metastasis, and lateral LN metastasis. Normality was assessed using the Kolmogorov-Smirnov test. Chi-square analysis was used to analyze categorical variables and the student t-test was used to analyze continuous variables. ^1^H-NMR spectroscopic data were analyzed using the Mann-Whitney U test because the results of the Kolmogorov-Smirnov test were statistically significant (*p*-value <0.05), which indicated that the data did not follow a normal distribution.

For multivariate analysis of spectral data, Matlab R2012a (MathWorks, Natick, MA), SIMCA-P version-13.0 software (Umetrics, Sweden), and Excel (Microsoft, Seattle, WA) programs were used. The spectral data were normalized to the total spectral area. The spectral region between 0.5 and 10 ppm was divided into bins of 0.01 ppm width. The water region from 4.6 ppm to 4.9 ppm was excluded prior to analysis. The binned data were aligned using the icoshift algorithm in Matlab [36], and were converted to the SIMCA-P format in Excel. Pareto scaling was used to preprocess the data. The intensity of each metabolite was normalized to the total intensity before statistical analysis. Orthogonal projections to latent structure discriminant analysis (OPLS-DA) is one of the popular methods for multivariate analysis in metabolomics [37]. Before OPLS-DA was performed, data were variable stability (VAST) scaled, with the standard deviation and the variation coefficients of the metabolites as scaling factors [38]. OPLS-DA were performed to maximize the separation between the two groups of interest. Statistical analyses were conducted using statistical software (R, Statistical Package version 3.3.3; R Foundation for Statistical Computing, Vienna, Austria; www.R-project.org). The muma package was used to perform OPLS-DA [39].

## Results

Tissue samples were available from 52 patients during the study period. The median age of the patients was 41.5 years (range 15–74 years). Twelve patients were male and 40 were female. The median tumor size was 23 mm (range 13–40 mm). Among the patients, 32 had central LN metastasis and 19 had lateral LN metastasis. All patients with lateral LN metastasis had central LN metastasis as well, thus the results of comparison in two ways, according to the presence or absence of LN metastasis and central LN metastasis were identical. Therefore the comparisons were analyzed in two ways according to the presence or absence of LN metastasis or the presence or absence of lateral LN metastasis. Four patients had distant metastasis.

No clinical or histopathological characteristic was significantly different for all comparisons when the patients were classified into two groups according to the presence or absence of LN metastasis or the presence or absence of lateral LN metastasis. Patient demographics and histopathological characteristics are shown in Table 1.

**Table 1:** Patient demographics and clinicopathologic characteristics.

Variable	Total	LN metastasis		*P*-value	Lateral LN metastasis		*P*-value
		(+)	(-)		(+)	(-)	
Number of patients	52	32 (61.5%)	20 (38.5%)		19 (36.5%)	33 (63.5%)	
Age	41.5 (15–74)	37 (15–66)	42.5 (16–74)	0.328	35 (15–60)	43 (16–74)	0.139
Gender							
Male	12 (23.1%)	7	5	0.795	6	13	0.270
Female	40 (76.9%)	25	15		6	27	
Primary tumor size (mm)	23 (13–40)	23 (13–40)	23 (14–37)	0.563	20 (13–40)	23 (14–37)	0.895
Distant metastasis	4 (7.7%)	4	0	0.100	4	0	0.014

Note–Unless otherwise specified, the data are the medians (range).

LN, lymph node.

^1^H-NMR spectroscopy quantified 24 metabolites in normal and PTC tissues. On univariate analysis, no metabolite showed significant difference between the two groups classified according to the presence or absence of LN metastasis or the presence or absence of lateral LN metastasis (Table 2).

**Table 2:** Comparison of metabolites obtained by ^1^H-NMR spectroscopy between patient groups classified by the presence of lymph node metastasis and lateral lymph node metastasis.

Metabolite concentration (mM)	LN metastasis		*P*-value	Lateral LN metastasis		*P*-value
	(+)	(-)		(+)	(-)	
Isoleucine	0.02 (0.01–0.47)	0.03 (0.00–0.47)	0.821	0.02 (0.01–0.16)	0.02 (0.00–0.47)	0.924
Leucine	0.05 (0.01–0.16)	0.06 (0.01–1.02)	0.880	0.05 (0.01–0.41)	0.05 (0.01–1.02)	0.761
Valine	0.06 (0.02–0.34)	0.06 (0.02–0.79)	0.940	0.05 (0.02–0.34)	0.06 (0.02–0.80)	0.642
Lactate	1.70 (0.61–10.51)	1.57 (0.39–7.11)	0.328	1.66 (0.61–10.51)	1.70 (0.39–7.11)	0.500
Threonine	0.07 (0.04–2.97)	0.08 (0.03–0.97)	0.873	0.07 (0.04–2.97)	0.08 (0.03–0.97)	0.518
Alanine	0.15 (0.04–0.58)	0.14 (0.03–1.50)	0.940	0.13 (0.04–0.58)	0.16 (0.03–1.50)	0.635
Uracil	0.02 (0.01–0.09)	0.02 (0.01–0.12)	0.461	0.03 (0.01–0.09)	0.16 (0.01–0.12)	0.218
Lysine	0.07 (0.02–0.56)	0.10 (0.01–1.15)	0.918	0.12 (0.02–0.56)	0.06 (0.01–1.15)	0.337
Glutamate	0.25 (0.08–2.51)	0.29 (0.04–2.53)	0.665	0.25 (0.10–0.56)	0.25 (0.04–2.53)	0.601
Methionine	0.04 (0.01–0.16)	0.04 (0.01–0.25)	0.397	0.04 (0.01–0.16)	0.04 (0.01–0.25)	0.655
Aspartate	0.07 (0.02–0.52)	0.06 (0.01–0.67)	0.288	0.07 (0.02–0.52)	0.07 (0.01–0.67)	0.464
Choline	0.03 (0.01–0.22)	0.03 (0.01–0.19)	0.714	0.03 (0.01–0.22)	0.03 (0.01–0.19)	0.635
Phosphocholine	0.19 (0.04–1.04)	0.20 (0.05–0.72)	0.925	0.21 (0.04–0.99)	0.19 (0.04–1.04)	0.655
Glycerophosphocholine	0.06 (0.02–616.00)	0.07 (0.02–0.25)	0.880	0.06 (0.03–0.41)	0.06 (0.02–616.0)	0.842
Taurine	0.55 (0.16–1.58)	0.50 (0.08–2.39)	0.763	0.52 (0.24–1.58)	0.50 (0.08–2.39)	0.512
Myo-inositol	1.21 (0.27–4.71)	1.40 (0.24–4.25)	0.652	1.14 (0.27–4.71)	1.25 (0.24–4.25)	0.798
Glycine	0.14 (0.04–0.98)	0.14 (0.04–2.06)	0.585	0.11 (0.04–0.98)	0.15 (0.04–2.06)	0.992
Phosphoethanolamine	0.35 (0.05–0.1.88)	0.54 (0.11–1.61)	0.560	0.31 (0.11–1.88)	0.44 (0.05–1.61)	0.992
Inosine	0.02 (0.00–0.13)	0.02 (0.00–0.12)	0.301	0.02 (0.00–0.13)	0.02 (0.00–0.13)	0.909
Tyrosine	0.02 (0.00–0.16)	0.02 (0.00–0.44)	0.893	0.02 (0.01–0.16)	0.02 (0.00–0.44)	0.270
Hypoxanthine	0.02 (0.00–0.16)	0.04 (0.01–0.51)	0.293	0.03 (0.01–0.16)	0.02 (0.00–0.51)	0.585
Formate	0.20 (0.01–0.89)	0.13 (0.00–0.48)	0.185	0.19 (0.05–0.89)	0.15 (0.00–0.48)	0.275
Succinate	0.02 (0.01–0.76)	0.03 (0.00–0.52)	0.297	0.02 (0.01–0.76)	0.02 (0.00–0.52)	0.790
Uridine	0.01 (0.00–0.02)	0.01 (0.00–0.02)	0.917	0.01 (0.00–0.02)	0.01 (0.00–0.23)	0.551

Note–Unless otherwise specified, the data are the medians (range).

LN, lymph node.

**Fig 1:**
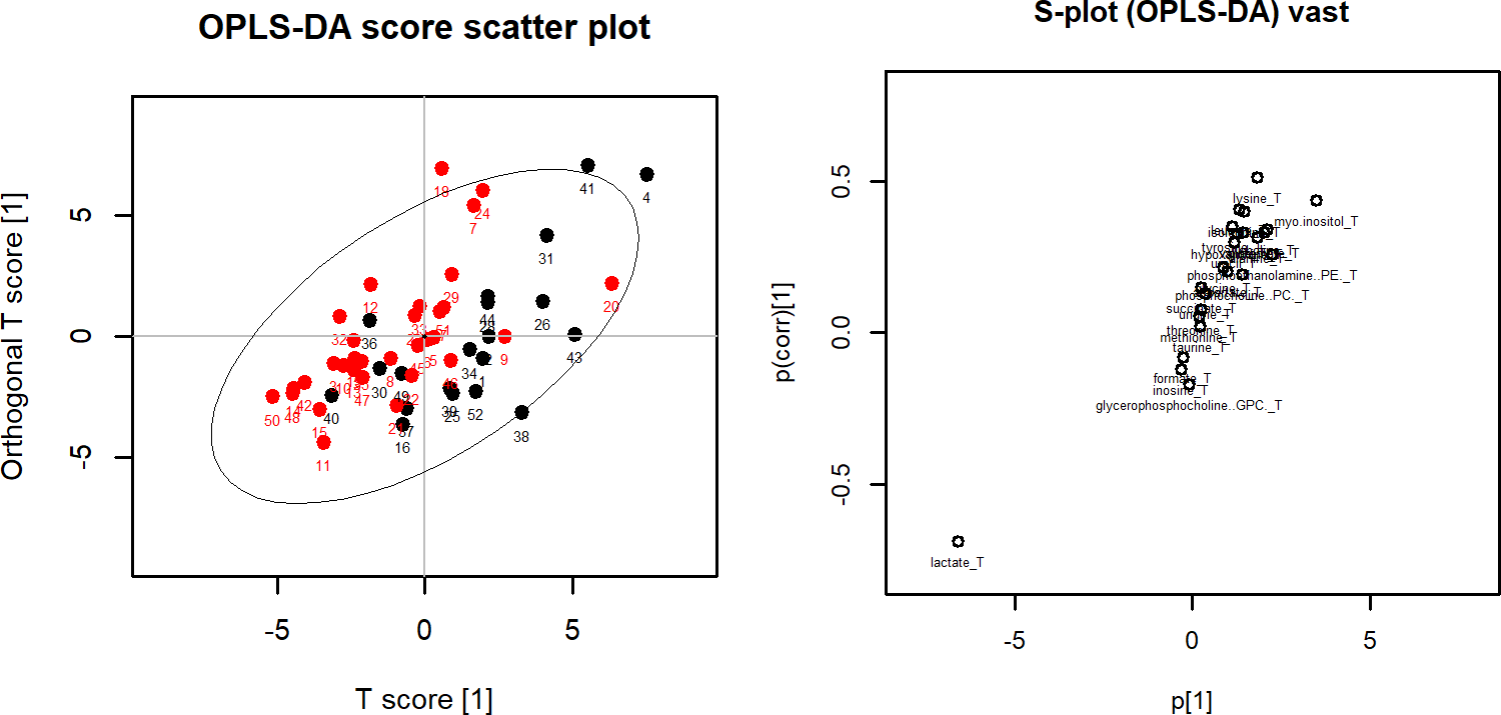
(Left) OPLA-DA score plot for lymph node metastasis. Red dots represent patients with lymph node metastasis and black dots represents patient without lymph node metastasis. The x-axis is the first component from OPLS-DA and the y-axis is the corresponding orthogonal score. (Right) OPLS-DS loading S-plot for lymph node metastasis. The x-axis is the covariation and the y-axis is the corresponding orthogonal score. The metabolites situated at the upper right or lower left sections are statistically relevant and represent possible discriminating variables.

**Fig 2:**
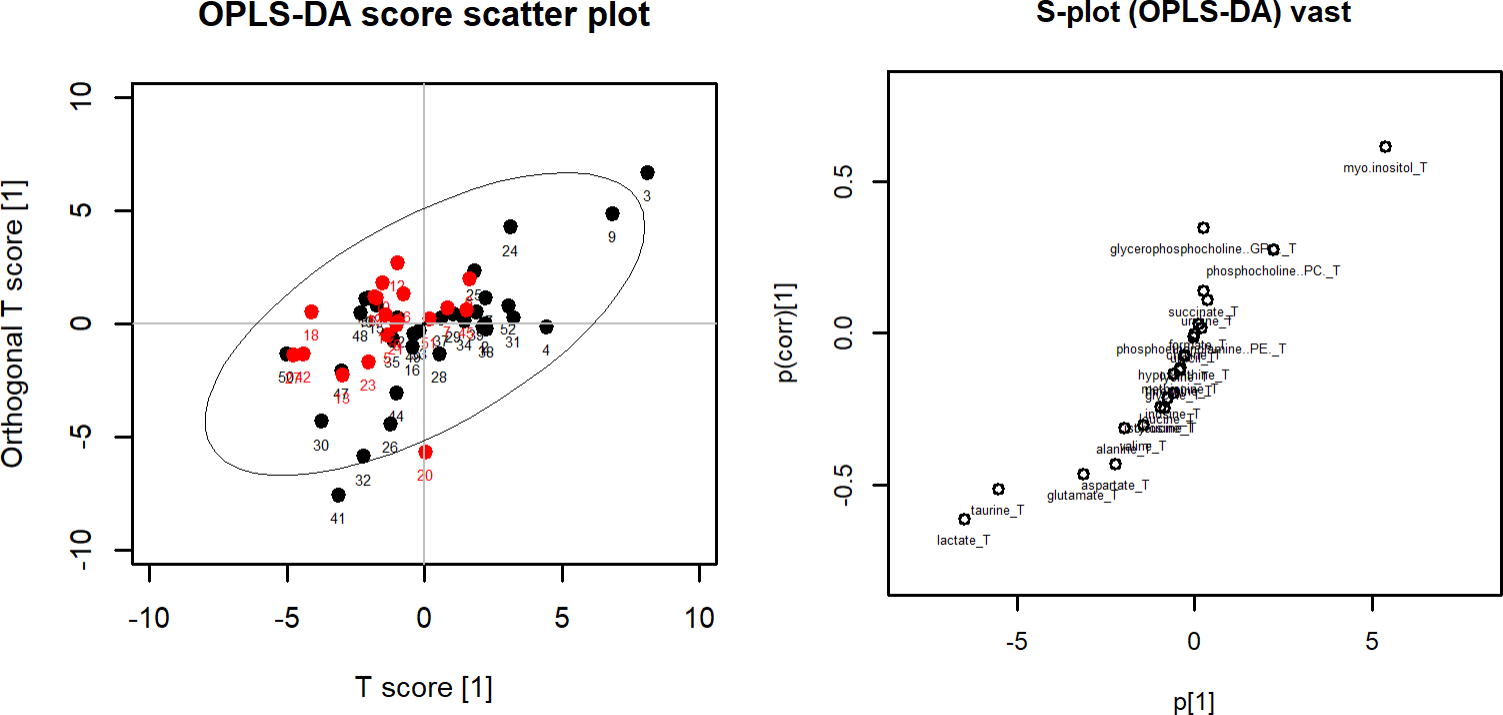
(Left) OPLA-DA score plot for lateral lymph node metastasis. Red dots represent patients with lateral lymph node metastasis and black dots represents patient without lateral lymph node metastasis. The x-axis is the first component from OPLS-DA and the y-axis is the corresponding orthogonal score. (Right) OPLS-DS loading S-plot for lateral lymph node metastasis. The x-axis is the covariation and the y-axis is the corresponding orthogonal score. The metabolites situated at the upper right or lower left sections are statistically relevant and represent possible discriminating variables.

OPLS-DA was performed to separate patients into two groups for each comparison. OPLS-DA score plots did not separate the two groups clearly for all three comparisons. When patients were classified according to the presence or absence of LN metastasis and central LN metastasis, the OPLS-DA score plot exhibited nonseparation between the two groups (A in Fig 1). The corresponding OPLS-DA loading S-plot showed lactate which was located in the left lower section of the S-plot as the most important metabolite to discriminate the two groups (B in Fig 1). When patients were classified according to the presence or absence of lateral LN metastasis, the OPLS-DA score plot exhibited nonseparation between the two groups (A in Fig 2) and the corresponding OPLS-DA loading S-plot showed that lactate in the left lower section of the S-plot and myo-inositol in the right upper section were the most important metabolites to discriminate the two groups (B in Fig 2).

## Discussion

The aim of this study was to explore metabolic differences in PTC according to the presence or absence of LN metastasis. We found that no metabolite could discriminate the two groups. However, lactate was found to be the most promising metabolite for discrimination.

Metabolomics is the analytic study of the metabolome, which differs in cancer cells and represents alteration of metabolic processes, and understanding the metabolome will allow deeper understanding of carcinoma development [27]. Several previous studies have applied metabolomics to PTC. In earlier times, studies were conducted to identify metabolic differences between thyroid neoplasms and normal thyroid tissue [34]. Normal thyroid tissue presented a higher level of lipids as well as lower levels of alanine, lactate and choline compared to neoplastic tissue [34].

Subsequent studies focused on discriminating benign and malignant thyroid neoplasms such as follicular adenoma or goiter nodules [40,41]. Recent studies further revealed that NMR spectroscopy could be applied to percutaneous FNA samples [42,43]. In these studies, malignant thyroid nodules were found to show higher relative concentrations of lactate and choline [43]. The results indicated that the NMR spectra of FNA cytology samples were similar to those of surgical specimens; hence, it had the potential to detect and classify thyroid tumors before surgery [42]. Furthermore, there was an attempt to discriminate nodular thyroid disease by analyzing urine and serum using ^1^H-NMR spectroscopy [44]. In this study, metabolomics could discriminate healthy controls from non-neoplastic nodules, follicular adenoma and PTC [44]. Increased lactate levels were observed in the blood serum of patients with nodular thyroid disease compared to healthy controls [44].

To our knowledge, this was the first research to discriminate the presence of metastatic LN in patients with PTC using ^1^H-NMR spectroscopy. Although our results failed to discriminate patients with and without LN metastasis, our data suggested the possibility of lactate being the most promising metabolite to predict LN metastasis. Lactate has been previously reported to increase in cancer [45]. Lactate reflects two important characteristics of biological changes that occur in tumor metabolism. First, tumor hypoxia shifts cellular energy production toward glycolysis from which lactate is generated as a by-product [45]. Second, it reflects aerobic glycolysis which tumors exhibit even if oxygen is present [46]. The importance of lactate is that it may indicate a more aggressive tumor phenotype that expresses LN metastasis in cancers of other organs [45,47]. In this study, lactate stood out as the most promising metabolite to represent the group with LN metastasis [47].

There are some limitations to this study. First, it is a retrospective study. Second, we performed ex vivo spectroscopy using surgical specimens. In vivo ^1^H-NMR spectroscopy is the most optimal diagnostic method for the preoperative diagnosis of thyroid nodules and prediction of prognosis. However, as performing in vivo ^1^H-NMR spectroscopy can be complicated by various issues such as thyroid movement during respiration or shimming difficulty due to large susceptibility differences between the neck and air in trachea, we decided to perform ex vivo ^1^H-NMR spectroscopy [48]. Third, our study was done with a small sample size and the proportion of patients with LN metastasis was relatively high which may explain the failure to discriminate LN metastasis.

In spite of these limitations, our data suggest that lactate may be used to predict LN metastasis and prognosis. Further studies with larger sample sizes are needed to elucidate significant metabolites which can indicate the presence of LN metastasis in patients with PTC.
